# Metagenomic analysis revealed the association between gut microbiota and different ovary responses to controlled ovarian stimulation

**DOI:** 10.1038/s41598-024-65869-6

**Published:** 2024-06-28

**Authors:** Xinyan Fo, Mei-li Pei, Pei-jun Liu, Feng Zhu, Yudan Zhang, Xin Mu

**Affiliations:** 1 Center for Translational Medicine, The First Affiliated Hospital of Xi’an Jiaotong University, No. 277 Yanta West Road, Xi’an, 710061 Shaanxi People’s Republic of China; 2 The Assisted Reproductive Medicine Center, Northwest Women’s and Children’s Hospital, No. 1616, Yanxiang Road, Xi’an, 710061 Shaanxi People’s Republic of China; 3 Department of Gynecology and Obstetrics, The First Affiliated Hospital of Xi’an Jiaotong University, No. 277 Yanta West Road, Xi’an, 710061 Shaanxi People’s Republic of China

**Keywords:** Metagenomic analysis, Gut microbiome, Ovary responses, FOI, Controlled ovarian stimulation, Microbiology, Diseases, Endocrinology, Health care, Medical research, Risk factors

## Abstract

The aim of this study was to assess the correlation between gut microbial taxonomy and various ovarian responses to controlled ovarian stimulation. A total of 22 IVF cycles with a follicle-to-oocyte index (FOI) < 0.5 and 25 IVF cycles with FOI ≥ 0.5 were included in this study. Baseline demographic characteristics were compared between the two groups. Metagenomic sequencing was performed to analyze fecal microbial community profiles. Mice were used to evaluate the effect of Bifidobacterium_longum on ovarian response to stimulation. Compared with FOI < 0.5 group, women in group with FOI ≥ 0.5 had significant more oocytes retrieved (p < 0.01). *Prevotella_copri, Bateroides_vulgatus, Escherichia_coli* and *Bateroides_stercoris* were more abundant in FOI < 0.5 group while *Bifidobacterium_longum, Faecalibacterium_prausnitzii, Ruminococcus_gnavus* and *Bifidobacterium_pseudocatenula* were more abundant in FOI ≥ 0.5 group. After adjusting for women’s age and BMI, Pearson correlation analysis indicated alteration of gut microbiome was related with serum E2, FSH, number of oocytes retrieved and clinical pregnancy rate. Animal study showed ovarian response will be improved after *Bifidobacterium_longum* applied*.* An increased abundance of *Bacteroidetes* and *Prevotella copri*, as well as a decreased abundance of *Bifidobacterium longum*, have been found to be associated with poor ovarian responsiveness. Changes in gut microbiomes have been observed to be correlated with certain clinical characteristics. The potential enhancement of ovarian response may be facilitated by the integration of *Bifidobacterium longum*.

## Introduction

Infertility was defined as a disease characterized by incapable of conceive after 1-year regular unprotected sexual intercourse^1^. The rate of infertility increased gradually due to different reasons^2^, affected 8–12% of spouses of childbearing age^3^. As an effective way to help achieving pregnancy, in vitro fertilization (IVF) had undergone fast development for more than 40 years^4^. Controlled ovarian stimulation (COS) was a crucial step in IVF. Different protocols were applied for ovarian stimulation, such as classical GnRH-a protocol, mild stimulation, antagonist protocol and so on. Conventionally, responsiveness to ovarian stimulation was classified as poor, suboptimal, normal or hyper-responders according to the oocyte numbers retrieved^5^. In 2018, Carlo Alviggi et al.^6^. illustrated a new marker named follicle-to-oocyte index (FOI) as a quantitative and independent indicator of ovarian response to stimulation, which would be closely related to the outcomes of IVF. Despite the large progress in this field, lower or absent ovarian response would happen in 8% of patients, which was unexpected and stressful to them^7^. In 2018, the Follicle-to-oocyte index (FOI), meaning the ratio between the total number of oocytes collected at the end of COS and the number of antral follicles available at the start of stimulation, was proposed to predict ovarian resistance to gonadotropin stimulation^6^. Many factors would contribute to poor ovarian response, including advanced women’s age, obesity, ovary surgery, previous chemotherapy or radiotherapy, pelvic adhesion and endometriosis^8–11^. However, the exact mechanism for poor ovarian response was still not fully understood.

Ovary was a common target organ for autoimmune antibody. The attack of autoimmune antibody to ovary would cause a kind of diseases including premature ovarian failure, polycystic ovarian syndrome and endometriosis^12^. The mechanism of these disorder pathologies remained obscure. Microbiota could be a possible reason. Changes in the diversity of the microbiome can be influenced by various environmental factors, including diet, lifestyle, medications, and genetic predispositions. These alterations may lead to the lost of immune tolerance, prolonged infection, impaired immune system function in the host, and ultimately contribute to the development of autoimmune diseases^13^.

Gut microbiome (GM) was a group of microorganisms existing in human gastrointestinal tract. It played complex effect on human health and diseases. For women, dysregulation of gut microbiome would be related to infertility via different pathway, including disturbing sex hormone, immune system and genital tract microbiome^14–16^. It was reported that dys-biotic of gut microbiome would be related to polycystic ovary syndrome, obesity, insulin resistance, impaired endometrium receptivity and premature ovarian insufficiency^17–20^. Recently, a series of studies indicated that gut microbiome would play some roles in development and maturation of host immune system, especially in autoimmune diseases^21–23^. Peptides derived from microbiome combined with human genetics, could make immune cells activated^24^. Disturbed microbiota and their derivatives might trigger the aberrant activation of innate immune cells, which leads to the upregulation of proinflammatory cytokine and reduction of anti-inflammatory cytokines^25^. These evidences indicated the possible relationship between gut microbiome and ovarian response to stimulation.

Metagenomics is a technique utilized to investigate the genetic composition of microbial communities without the need for isolating individual microorganisms. This method enables a comprehensive exploration of microbial diversity, functionality, and their ecological significance, particularly for microorganisms that were previously challenging or impossible to culture. In recent years, Metagenomic Sequencing has been widely used in microbiology research, spanning from basic research to clinical practice, including the factors influence the composition and diversity of human gut microbiota, the dynamic changes that occur in the human microbiota in the days and months after birth and the global ocean microbiome^26^. In female, an intimate association was found to link microbial dysbiosis with the pathophysiologic changes of PCOS^27^. microbiota could be dissected and applied in the treatment of ovarian cancer based on a predictive, preventive, and personalized medicine (PPPM) model^28^.

However, until now, there was few studies linking gut microbiome with different ovarian responses to controlled ovarian stimulation. The objective of this study was to profile gut microbiome community of different ovarian responsiveness. We aimed to conduct a metagenomic gene sequencing analysis of fecal samples and analyze the correlations between the gut microbiota and different ovarian response.

## Materials and methods

### Study populations

This retrospective case–control study was conducted at our center from January 2022 to July 2022. All the patient’s data were extracted from the electronic medical data system. The study protocol was approved by the Ethics Committee for the Clinical Application of Human Assisted Reproductive Technology of Northwest Women’s and Children’s Hospital (No. 2022002). Written informed consent were obtained from every patient participated in this study and the study was performed in accordance with the Declaration of Helsinki.

All the patients met the inclusion criterions were considered eligible. The inclusion criterions were as follows: (1) women who underwent their first fresh IVF cycle; (2) under 35 years old; (3) with body mass index (BMI) under 30 kg/m^2^; (4) using gonadotropin releasing hormone agonist (GnRH-a) long protocol for controlled ovarian stimulation; (5) willing to have blood drawn an donate fecal sample at the start of pituitary downregulation. The exclusion criterions were: (1) women who refused to have blood drawn or provide fecal sample; (2) had chronic pelvic or ovarian inflammation, endometriosis, autoimmune diseases; (3) with polycystic ovary syndrome (PCOS); (4) a history of major gastrointestinal surgery within 3 years; (5) a history of severe gastrointestinal disease, especially inflammatory bowel disease; (6) use of oral probiotics within 1 month; (7) use of antibiotic drug within a month. All the women were divided into two groups according to the FOI. Low ovarian sensitivity or hypo-responsiveness was denoted by FOI < 0.5.

### Controlled ovarian stimulation

All the women received daily injection of 0.1 mg triptorelin for pituitary downregulation from mid-luteal phase of menstrual cycle. We simultaneously draw venous blood from the patients for sex hormone testing. Fourteen days later, when the pituitary downregulation was completed, the gonadotropin (Gn 75-300U/d) was administrated for ovarian stimulation. The process lasted for almost 10–15 days. The dosage was adjusted according to the women’s BMI, age hormone level and response to drug. Transvaginal ultrasound was used every 2–5 days to evaluate the follicle growth. When two follicle diameters reached 18 mm or three follicle diameters reached 17 mm, human chorionic gonadotropin (HCG) was applied for triggering final follicle maturation. Thirty-six hours later, oocyte retrieve was performed.

### Fecal sample collection and preparation

Fecal specimens were collected within 3 days before the start of pituitary downregulation by the participants in hospital or at home using fecal sample collection tube. If possible, the sample was delivered in a cooler on the day of collection. If not possible, the sample was stored at − 20 °C for 1 or 2 days until delivery. Then the aliquot was prepared and stored at − 80 °C until further analysis.

### Metagenomic sequencing

Bacterial DNA extraction and metagenomic sequencing were performed with standard procedures as previously described. Briefly, the Magbeads Fast DNA^®^ kit (MP Biomedicals, Shanghai, China) was used following the manufacturer’s protocol to extract bacterial DNA from the stool samples. NanoDrop 2000 was used to measure the concentrations of the extracted DNA, followed by 1% agarose gel electrophoresis for integrity detection using KAPA HyperPlus PCR-free reagent (Illumina, CA, US). DNA was trimmed to an average of approximately 400 bp to construct a paired-end library. Qubit 2.0 (Thermo Fisher, Massachusetts, US) was used to measure DNA quantity, and the reads quality control procedure was conducted using Agilent 2100 (Agilent, Palo Alto, US). Then, pooling was performed on all sample libraries, and the total amount of DNA after pooling was required to be 300 ng, with a volume of 48 µl. After heat denaturation, single-strand circularization, enzyme digestion, and purification, shotgun metagenomic sequencing was performed by Shaan Probiomicros Co.,Ltd. (Xi’an, China) on the BGI platform (MGESEQ-T7, Shenzhen, China). Use fastp (v0.23.0) (length_required = 50, n_base_limit = 2) to remove low-quality reads and adaptors from the raw reads, and use Bowtie2 software (v2.3.5.1) to exclude the host reads based on the human genomes 38.

### Species analyze

MetaPhlAn4 is a tool for analyzing the composition of microbial communities (bacteria, archaea, eukaryotes, and viruses). In this project, MetaPhlAn4 software was used to annotate the metagenomic data and analyze the composition of microbial communities to obtain relative abundance information. Species abundance and species composition were visualized using stack maps, heat maps, pie charts, et al.

Microbial alpha-diversity was measured using Shannon, Simpson, and InvSimpson index. Significant differences in alpha-diversity indexes between cases and controls were determined using the Wilcoxon test. Differences in overall communities (beta-diversity) were calculated using the Bray–Curtis distance metric and visualized by Principal Component Analysis (PCA), Principal Co-ordinates Analysis (PCoA), and Non-metric multidimensional scaling (NMDS). Significant differences across groups were evaluated using permutational multivariate analysis of variance (PERMANOVA).

### Bacterial culture

As the relative abundance of Bifidobacterium longum was highest in FOI ≥ 0.5 group. We choose Bifidobacterium longum to validate in vitro experiments. The strain of Bifidobacterium longum was procured from a commercial source (Shaan Probiomicros Co.,Ltd, Xi’An, China) and was subsequently cultured in nutrient-rich liquid PYG (Peptone, Yeast Extract, and Glucose) medium. The culturing process was conducted under anaerobic conditions for a duration of 24 h at a constant temperature of 37 °C. Post-culturing, the samples were preserved at a temperature of − 20 °C until further use.

### Animal study

A cohort of twenty female C57 mice, aged 4 weeks, were obtained from Beijing Vital River Laboratory Animal Technology (Beijing, China). They were all housed under a controlled environment with a 12-h light/dark cycle at a constant temperature and humidity and were provided with ad libitum access to food and water at the Animal Research Center of the First Affiliated Hospital. Subsequently, the mice were randomly allocated into two distinct groups: a control group and a Bifidobacterium longum group. In the Bifidobacterium longum group, the bacteria were resuspended in saline, with each mouse receiving over 1 × 10^9^ CFU of the bacteria via gavage, administered daily for a period of 30 days. Meanwhile, in the control group, the mice were administered with PBS resuspended in saline via gavage daily for the same duration. On the 30th day of the experiment, all the mice were intraperitoneally injected with 10 IU of PMSG, followed by an administration of hCG (10 IU) 48 h subsequently. Thirteen hours post the hCG treatment (between 8 and 9 a.m.), the mice were euthanized under anesthesia through cervical dislocation. The ovulated cumulus-oocyte complex (COCs) was then extracted from the oviduct. After undergoing three rinses in M2 media, which contained 0.5 mg/mL of hyaluronidase, the oocytes were collected and quantified under a somatic microscope and those with a discernible polar body were categorized as mature (Metaphase II, MII). The animal study was approval by the Ethics Committee of the First Affiliated Hospital of Xi’an Jiaotong University(No: 2022-497) and all experiments were performed in accordance with ARRIVE guidelines.

### Statistical analysis

All the clinical data were analyzed by SPSS software package v.27.0 (IBM, NY, USA). Kolmogorov–Smirnov test was used to evaluate whether the distribution was normality. For continuous variables, Student’s t-test or Mann–Whitney U test was employed (presented as mean ± SD). Gut microbiome analyses were performed using R software (R-4.3.1). Unordered categorical variables were analyzed by Fisher’s exact test and expressed as counts and proportions. The Wilcoxon test (corrected by the Benjamini–Hochberg method, false discovery rate < 0.05) was used to detect the difference in relative abundance between groups. All *P* values < 0.05 were considered statistically significant. Pearson correlation analysis was performed for evaluating correlations between clinical data and differential gut microbiome. Receiver operating characteristic (ROC) curve was applied to evaluate the diagnostic efficacy and accuracy of gut microbiome. The methods and R packages used in the project are shown in the Table 1.

**Table 1:** Methods and R package in the project.

Method	R. package	Version
PERMANOVA	vegan	2.6-4
Alpha	vegan	2.6-4
MaAsLin2	Maaslin2	1.14.1
PCoA	vegan	2.6-4
RandomForest	randomforest	4.7-1.1

### Ethical approval

The study received approval by the Ethics Committee for the Clinical Application of Human Assisted Reproductive Technology of Northwest Women’s and Children’s Hospital. Informed consent was obtained from all individual participant include in the study.

## Results

### Comparison of baseline characters of two groups

A total of 47 IVF cycles were included in our study. Among them, there were 22 cycles in FOI < 0.5 group and 25 cycles in FOI ≥ 0.5 group. Baseline characters including maternal age, body mass index (BMI), antral follicle count (AFC), infertility duration, infertility type, follicle stimulation hormone (FSH), luteinizing hormone (LH), estrogen (E2) at the start of pituitary downregulation, Gn dose, Gn time, number of oocytes retrieved and clinical pregnancy rate were compared between two groups. Compared with FOI < 0.5 group, women in group with FOI ≥ 0.5 had significant more oocytes retrieved (*p* < 0.01). Additionally, a trend towards an increased clinical pregnancy rate was observed. There were no significant differences for other characters between two groups (Table 2).

**Table 2:** Demographic and laboratorial data between FOI < 0.5 and FOI ≥ 0.5.

Group	FOI < 0.5 (n = 22)	FOI ≥ 0.5 (n = 25)	*t/χ*^*2*^	*P*
Maternal age (years)	29.13 ± 4.43	30.60 ± 4.35	− 0.14	0.26
Maternal BMI (kg/m^2^)	21.69 ± 3.19	21.40 ± 2.38	0.36	0.72
Paternal BMI (kg/m^2^)	23.87 ± 2.92	23.22 ± 3.21	0.73	0.47
Duration of Infertility (years)	6.73 ± 4.15	6.72 ± 4.71	0.01	1.00
Basal AFC (n)	18.77 ± 3.04	16.68 ± 4.40	1.87	0.07
FSH (IU/L)	8.11 ± 2.09	7.38 ± 1.87	1.26	0.21
LH (IU/L)	14.25 ± 6.58	13.31 ± 7.76	0.44	0.66
E2 (pg/ml)	112.67 ± 51.69	85.83 ± 45.36	1.90	0.06
Gn dose (IU)	2007.95 ± 729.33	2148.00 ± 693.46	− 0.67	0.50
Gn time (day)	8.27 ± 2.76	8.96 ± 2.24	− 0.94	0.35
Number of retrieved oocytes (n)	7.73 ± 1.61	9.16 ± 2.75	− 2.14	**0.04**
Clinical pregnancy rate (%)	52.94 (9/17)	63.16 (12/19)	0.39	0.54

*p* values in bold indicate statistical significance (p ≤ 0.05).

FOI, follicle-to-oocyte index; IU/L, international units per milliliter; kg/m^2^, kilogram per m^2^; pg/ml, pg per ml; n, number;

### Characteristics of gut microbiome between two groups

**Figure 1:**
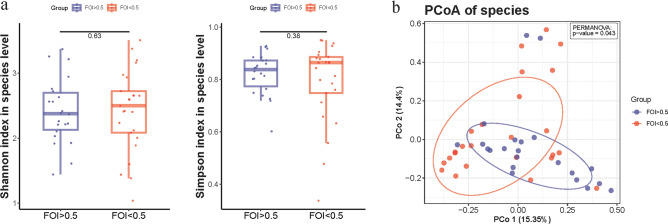
Overall differentiation of gut microbiota between two groups. (**a**) Shannon index in species level between two groups. (**b**) PCoA analysis between two groups.

First, through Metaphlan (v4.0.3), we identified gut microbiome taxa of all the samples at different levels. A total of 10 phyla, 22 classes, 37 orders, 70 families, 175 genu and 523 species were identified. Next, we analyzed differences in gut microbiome alpha and beta diversity between two groups. The results revealed that there was no difference between two groups in the alpha diversity which representing the richness and evenness of the bacterial community (*p*_Shannon_ = 0.63, *p*_InverseSimpson_ = 0.38). PCoA was performed based on UniFrac distance for beta diversity, which representing the dissimilarity of gut microbiome. The result showed significantly separation of the microbial composition at the species level between two groups (PERMANOVA *p* = 0.043) (Fig. 1).

**Figure 2:**
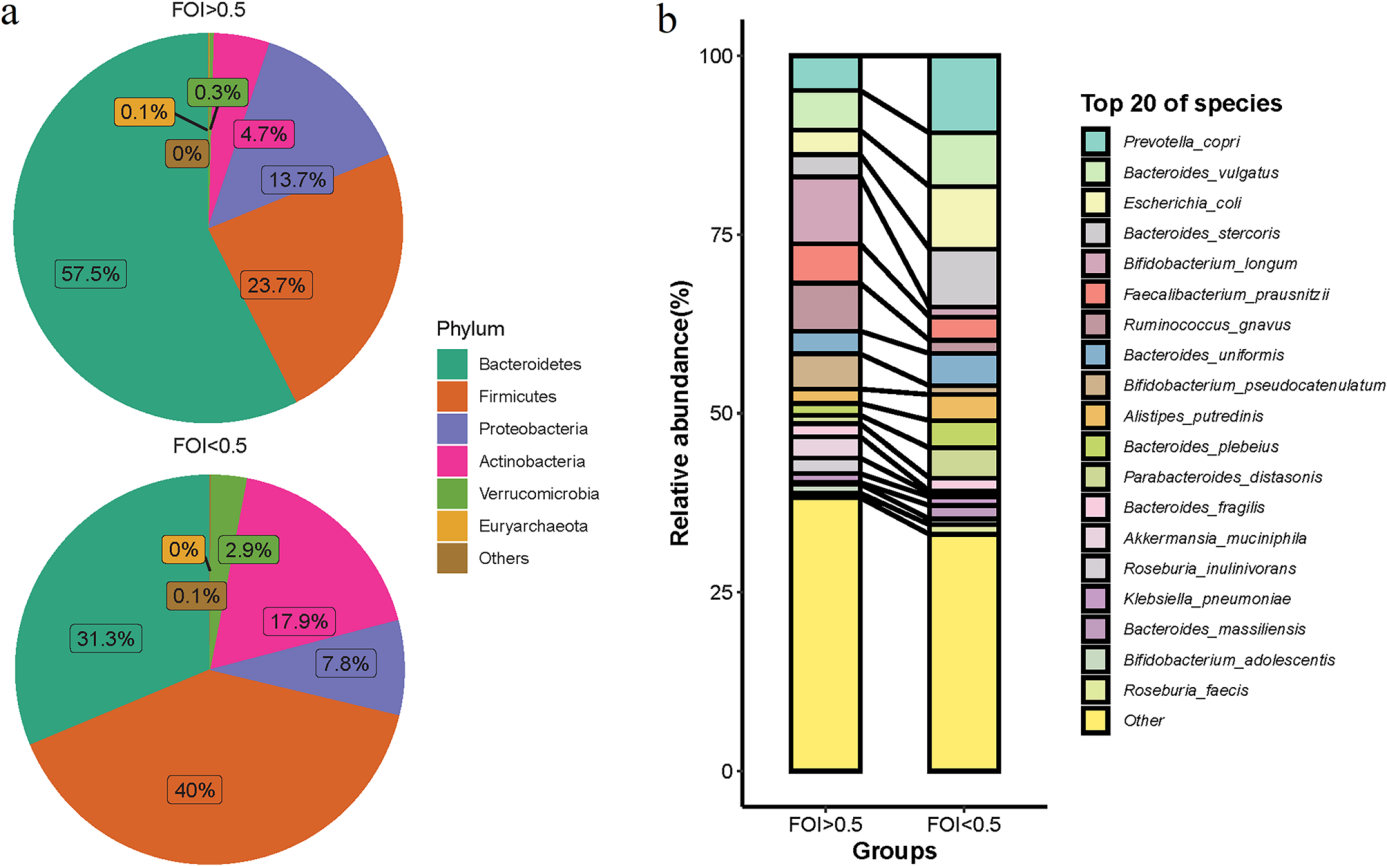
Microbial community profiles of gut microbiome between two groups. (**a**) Pie showed the relative abundance of the main phylum between two groups. (**b**) Top 20 relative abundance species between two groups.

Then, we analyzed the distributions of gut microbiome communities. We found that *Bacteroidetes*, *Firmicutes, Proteobacteria and Actinobacteria* were the dominate phyla in both FOI < 0.5 and FOI ≥ 0.5 group. *Bacteroidetes* was the predominant microbe in two groups. The percentage of *Bacteroidetes* was significantly higher in FOI < 0.5 group, accounting for 57.5% in FOI < 0.5 group while 31.3% in FOI ≥ 0.5 group, respectively (Fig. 2a). The top 20 abundant species of two groups were shown in Fig. 2b. *Prevotella_copri, Bateroides_vulgatus, Escherichia_coli and Bateroides_stercoris* were more abundant in FOI < 0.5 group while *Bifidobacterium_longum, Faecalibacterium_prausnitzii, Ruminococcus_gnavus* and *Bifidobacterium_pseudocatenula* were more abundant in FOI ≥ 0.5 group.

**Figure 3:**
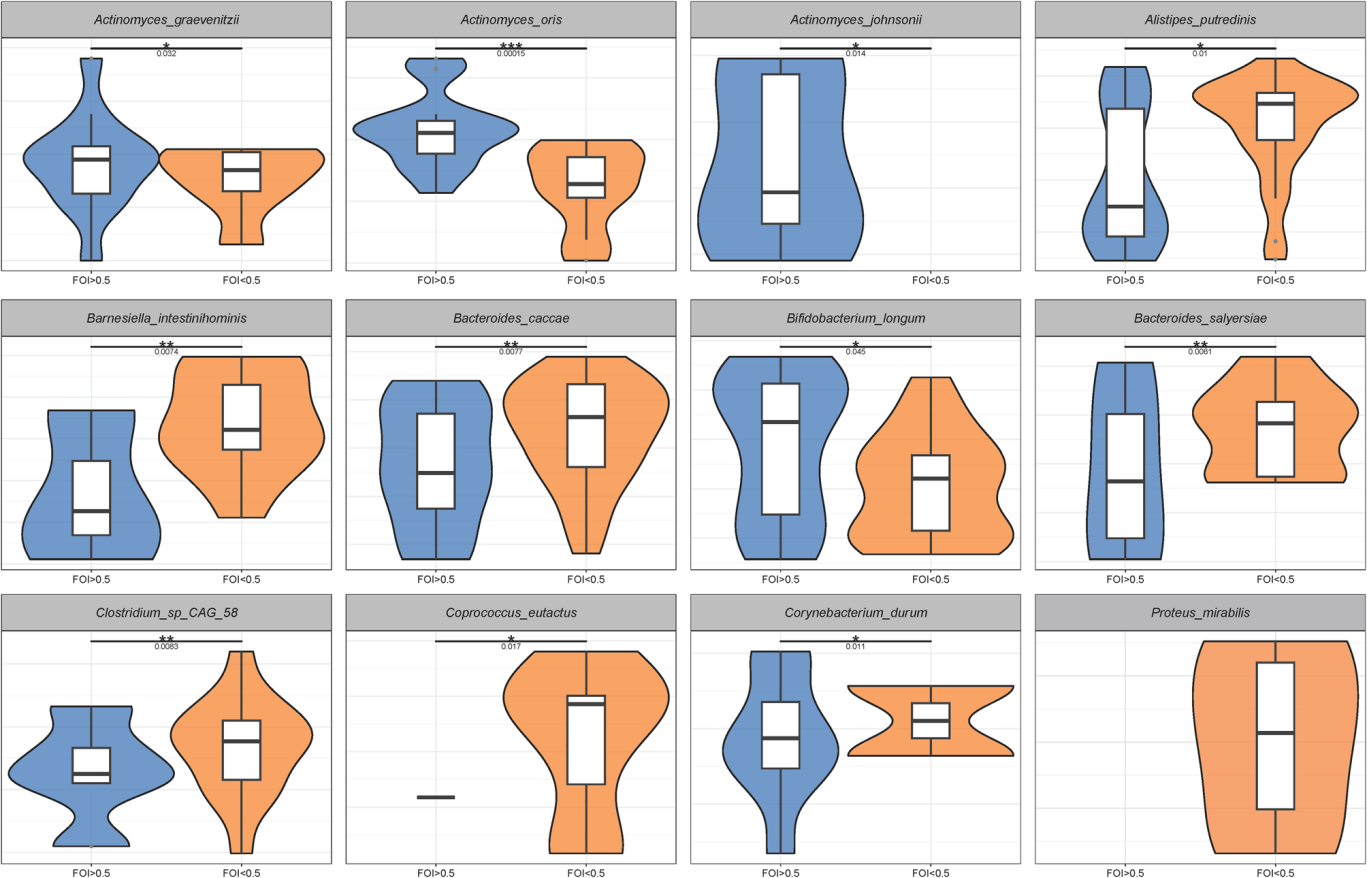
Box-plot showed the significantly different and important microbiomes between two groups at species level. The vertical axis showed the relative abundance of differential microbiomes.

We analyzed differential species between two groups with Wilcoxon Rank sum tests. Compared with FOI ≥ 0.5 group, *Bacteroides_caccae* (*p* = 0.0077), *Bacteroides_salyersiae* (*p* = 0.0061), *Alistipes_putredinis* (*p* = 0.01), *Barnesiella_intestinihominis* (*p* = 0.0074), *Clostridinm_sp_CAG_58* (*p* = 0.0083), *Coprococcus_eutactus* (*p* = 0.017), *Corynebacterium_durum* (*p* = 0.011) and *Proteus_mirabilis* (*p* < 0.001 ) increased significantly while *Bifidobacterium_longum* (*p* = 0.045), *Actinomyces_graevenitzii* (*p* = 0.032), *Actinomyces_johnsonii* (*p* = 0.014) and *Actinomyces_oris* (*p* = 0.00015) significantly decreased in FOI < 0.5 group (Fig. 3).

### Association between clinical characteristics and differential gut microbiome

**Figure 4:**
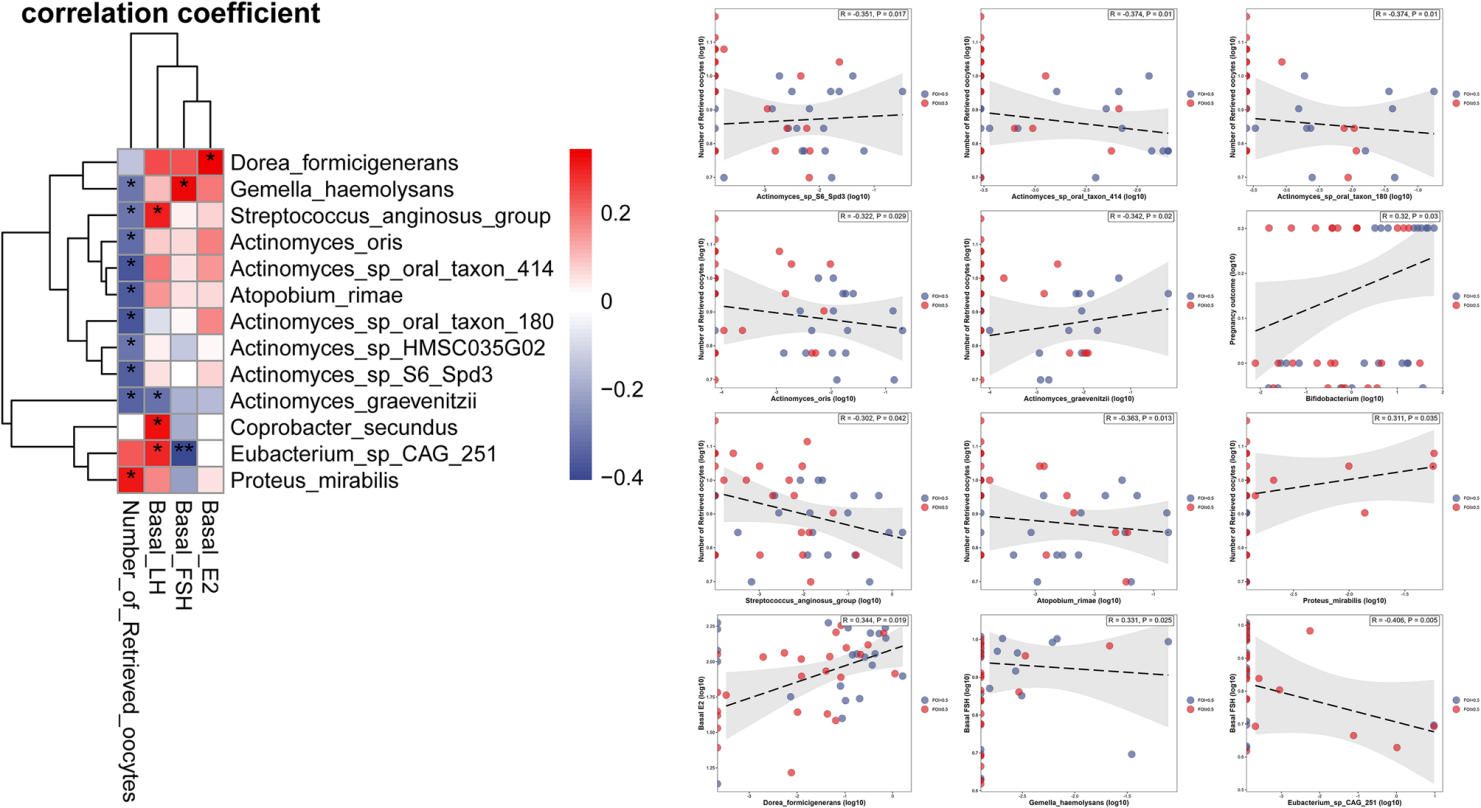
Association between gut microbiome and clinical characteristics. The left picture was the heatmap. The right pictures showed the exact correlation plot for serum E2, Serum FSH, number of oocytes retrieved, clinical pregnancy rate and gut microbiome. The vertical axis represented the clinical characteristics and the horizontal axis represented the gut microbiome.

To evaluate the association between differential gut microbiome and clinical characteristics, Pearson correlation analysis was performed. As age and BMI were important influencing factors that affect the composition and function of gut microbiota, we adjusted for maternal BMI and age in the analysis. The results showed that serum E2 level was positively related with *Dorea-formicigenerams*, serum FSH level was positively related with *Gemella-haemolysans* and negatively related with *Eubaterium-sp.CA-251*. Number of oocytes retrieved was positively related with *Proteus-mirabilis* but negatively related with six *Actinomyces species* and *Streptococcus-anginosus-group.* Clinical pregnancy rate was positively related with bifidobacterium (Fig. 4).

### Effect of *Bifidobacterium_longum* on ovarian response

In mice, a significant increase in the number and rate of metaphase II (MII) oocytes was observed in *Bifidobacterium_longum* group compared to the control group. Specifically, the total number of MII oocytes in the *Bifidobacterium_longum* group was 239, compared to 180 in the control group. Furthermore, the rate of MII oocytes was markedly higher in the *Bifidobacterium_longum* group (79.67%) compared to the control group (65.45%) (*p* < 0.05, Table 3).

**Table 3:** Comparison of oocytes between *Bifidobacterium_longum* and control group in mice.

Group	*Bifidobacterium_longum* group	Control group	χ^2^	*P*
Total oocytes	300	275		
MII number	239	180		
MII rate (%)	79.67%	65.45%	14.659	0.000

## Discussion

The imbalance of the gut microbiome can lead to immune and inflammation changes in the ovary. Previous studies have mainly focused on infertility diseases using 16S rRNA sequencing. In our study, we utilized metagenomic analysis to examine the community profile of different ovarian responses to stimulation. We discovered associations between clinical parameters and distinct gut microbiomes. Additionally, we conducted experiments on mice using microbiota to validate its role in the response to ovarian hyperstimulation.

Ovarian stimulation is a crucial step in the IVF process. Experiencing an unexpected lower response to stimulation can be a stressful event during IVF. According to the POSEIDON criterion, approximately 47% of patients undergoing Assisted Reproductive Technology (ART) are affected by low ovarian response^29,30^. The follicle-to-oocyte index (FOI), a quantitative marker, was utilized to assess the dynamic response of the ovary to ovarian stimulation (OS), with FOI < 0.5 typically indicating hypo-response. However, the exact mechanism underlying hypo-responsiveness to OS remains incompletely understood. It is possible that immunity and inflammation play a role in the pathogenesis^9,31,32^.

Our study showed that *Bacteroidetes, Firmicutes*, *Proteobacteria*, and *Actinobacteria* were the dominant phyla in both the FOI < 0.5 and FOI ≥ 0.5 groups, with *Bacteroidetes* being the predominant microbe in both groups. This finding is consistent with previous studies^33^. However, there was a significant difference in beta diversity between the two groups. The decrease in diversity in the FOI < 0.5 group will lead to a reduction in cell–cell junctions, an increase in gut permeability, and ultimately result in systemic inflammation^34^.

Bacteroidetes is one of the phyla that plays an important role in maintaining the homeostasis and health of the human intestine^35^. The toxin produced by *Bacteroides fragilis* is known to have pro-inflammatory properties and can trigger cross-reactive responses in collagen-induced arthritis^36,37^. Increases abundance of *Prevotella_copri* will display increased inflammation, was correlated with disease in new-onset untreated rheumatoid arthritis (NORA) patients^38^. Meanwhile, short-chain fatty acids (SCFAs), which was produced by some gut microbiome such as *Bifidobacterium,* could promote peripheral Treg cell generation^39^ and inhibit cytokine production by iNKT cells^40^.

*Bidiobacterium longum*, a stable taxon present in the human gastrointestinal tract, plays a role in immune regulation and anti-inflammation processes. In animal models, the combination of *B. longum* and *Lactobacillus* rhamnosus has a synergistic effect, enhancing IL-10 levels and improving periodontitis^41^. *B. longum subsp. Infantis B6MNI* has the ability to modulate the gut microbiota and fecal metabolites. It also has an impact on the expression of immune cell differentiation and can delay the progression of rheumatoid arthritis^42^. For patients with diarrhoea-predominant irritable bowel syndrome (IBS-D), a reduction in the levels of pro-inflammatory cytokines, and intestinal permeability as well as an improvement in gastrointestinal symptoms were observed after treating with *B. longum* ES1 for 8 or 12 weeks^43^. *B. longum* L556, isolated from healthy human feces, enhanced anti-inflammatory effects by modulating gut microbiota and metabolites like SCFAs, ultimately regulated lipid and amino acid metabolism in coronary heart disease group^44^.

As a significant member of the Firmicutes phylum, Faecalibacterium prausnitzii commonly constitutes up to 5% of the fecal microbiota in healthy individuals. As a significant member of the Firmicutes phylum, Faecalibacterium prausnitzii commonly constitutes up to 5% of the fecal microbiota in healthy individuals. This bacterium produces butyrate, which has been shown to reduce the severity of pathogenic bacterial infections, enhance mucosal barrier integrity, promote the dominance of obligate anaerobic bacteria, and decrease oxygen availability in the gut. Additionally, Faecalibacterium prausnitzii has been observed to mitigate excessive inflammation by modulating immune cells, such as enhancing the functions of M2 macrophages and regulatory T cells, inhibiting neutrophil infiltration, and suppressing the NF-κB pathway^45^. These actions play a critical role in maintaining intestinal homeostasis. In humans, Faecalibacterium prausnitzii is implicated in various immune and inflammation-related diseases, including systemic lupus erythematosus (SLE), atopic dermatitis, psoriasis, and HIV^46^.

In our study, we observed a significant increase in *Bacteroidetes* and *Prevotella_copri,* while *Bifidobacterium_longum* showed a significant decrease in the FOI < 0.5 group compared to the FOI ≥ 0.5 group. These findings suggest that inflammation and immune responses may play a role in different ovarian responses mediated by the gut microbiome.

After adjusting for age and BMI, our study found that serum sex hormones, including E2 and FSH, were correlated with certain gut microbiome profiles. These findings are consistent with previous studies^20,47^. The number of oocytes retrieved was also found to have a negative relationship with the *Streptococcus-anginosus*-group. This could be partially attributed to changes in the Estrogen-gut microbiome axis^33^. However, the precise mechanism remains not fully elucidated, necessitating further exploration and investigation.

In the present investigation, metagenomic sequencing was utilized for the identification of microbiota at the strain level, providing superior accuracy compared to 16S sequencing. Despite these advancements, the study was not without limitations. As an observational, cross-sectional, and single-center study, the sample size was insufficient for the construction of a diagnostic tool. Therefore, future research necessitates larger, meticulously designed prospective studies, supplemented with more animal experiments, to substantiate and fortify our hypotheses.

In conclusion, our research investigated the differential responses to ovarian stimulation within the gut microbiome community profile. It was observed that an increased abundance of *Bacteroidetes* and *Prevotella_copri*, along with a decreased presence of *Bifidobacterium_longum*, is associated with poor ovarian responsiveness. Variations in gut microbiomes were found to correlate with specific serum sex hormones and oocyte retrieval. Furthermore, supplementation with *Bifidobacterium longum* in mice markedly enhanced the response to ovarian hyperstimulation. Consequently, the modulation of gut microbiota may potentially serve as a therapeutic strategy for managing ovarian hypo-responsiveness to ovarian stimulation.

## Data Availability

The datasets analyzed during the current study available from the corresponding author on reasonable request.
